# A High Ductal Flow Velocity Is Associated with Successful Pharmacological Closure of Patent Ductus Arteriosus in Infants 22–27 Weeks Gestational Age

**DOI:** 10.1155/2012/715265

**Published:** 2012-12-18

**Authors:** Karl Wilhelm Olsson, Anders Jonzon, Richard Sindelar

**Affiliations:** Department of Women's and Children's Health, Uppsala University Children's Hospital, Uppsala University, SE-751 85 Uppsala, Sweden

## Abstract

*Objective*. To identify factors affecting closure of patent ductus arteriosus (PDA) in newborn infants born at 22–27 weeks gestational age (GA) during pharmacological treatment with cyclooxygenase inhibitors. *Method*. Infants born at 22–27 weeks of GA between January 2006 and December 2009 who had been treated pharmacologically for PDA were identified retrospectively. Medical records were assessed for clinical, ventilatory, and outcome parameters. Echocardiographic examinations during treatment were reviewed. *Results*. Fifty-six infants were included in the study. Overall success rate of ductal closure with pharmacological treatment was 52%. Infants whose PDA was successfully closed had a higher GA (25 + 4 weeks versus 24 + 3 weeks; *P* = 0.047), and a higher pretreatment left to right maximal ductal flow velocity (1.6 m/s versus 1.1 m/s; *P* = 0.023). Correcting for GA, preeclampsia, antenatal steroids, and age at start of treatment, a higher maximal ductal flow velocity was still associated with successful ductal closure (OR 3.04; *P* = 0.049). *Conclusion*. Maximal ductal flow velocity was independently associated with success of PDA treatment.

## 1. Introduction

Infants born before 28 gestational weeks have a high incidence of patent ductus arteriosus (PDA) [1, 2]. The postnatal presence of a haemodynamically significant left to right shunt through the duct is associated with a lower survival rate and an increased incidence of intraventricular haemorrhage (IVH), necrotizing enterocolitis (NEC), and bronchopulmonary dysplasia in preterm infants [3–9]. Inhibition of prostaglandin production with the cyclooxygenase inhibitors indomethacin or ibuprofen is the standard pharmacological treatment for PDA [10]. Nevertheless failure of pharmacologic treatment frequently occurs in extremely preterm infants and is associated with increased mortality [11–13].

Previous studies have identified low gestational age (GA), pregnancy-induced hypertension, antenatal indomethacin exposure, lack of antenatal glucocorticoid exposure, late indomethacin treatment, respiratory distress syndrome (RDS), use of high-frequency oscillatory ventilation (HFOV), large ductal diameter, and less ductal shunt velocity as independent risk factors for failure of pharmacological treatment of PDA [14–18].

Pulmonary factors such as prenatal steroid exposure and RDS thus appear to affect the closure of the ductus arteriosus, and parameters related to pulmonary circulation, for example, high PaO_2_ and low blood pressure within the ductus arteriosus, relate to physiological ductal constriction in animal studies [19]. The objective of this retrospective study was to identify factors associated with closure of the ductus arteriosus during treatment with cyclooxygenase inhibitors in infants born at 22–27 weeks GA, with special focus on ventilatory and pulmonary circulatory factors.

## 2. Patients and Methods

### 2.1. Patients

Infants born at Uppsala University Children's Hospital between January 2006 and December 2009 at a GA of less than 28 weeks and pharmacologically treated for PDA were included in this retrospective cohort study. Infants with any major congenital anomalies were excluded. The study was approved by the Swedish Central Ethical Review Board.

### 2.2. Pharmacological Treatment for Patent Ductus Arteriosus

All newborn infants born at a GA of less than 28 weeks were evaluated echocardiographically within the first days of life and echocardiographic examinations were repeated if indicated. Examinations were performed using an Acuson Sequoia Ultrasound System (Siemens AB, Upplands Väsby, Sweden) with a 10 MHz transducer and results were saved in digital format (Xcelera, Philips AB, Stockholm, Sweden).

A haemodynamically significant PDA was defined by one or more of the following echocardiographic parameters: ductus arteriosus diameter of >1.5 mm; left atrium to aortic root ratio (LA/Ao) of >1.5; reduced and reversed flow during diastole in the descending aorta in combination with clinical signs. Pharmacological treatment was initiated when a PDA of haemodynamic significance was found and none of the following contraindications were present: ductal-dependent heart defect; renal failure (serum creatinine >120 *μ*mol/L or serum urea >12 mmol/L); thrombocytopenia (platelets <50 × 10^9^/L); IVH grade II–IV; or NEC. Indomethacin (Indocid, Merck & Co., Inc., West Point, Pennsylvania, USA) was administered in a three-dose regimen as an infusion (0.2 mg/mL, 0.2 mg/kg/dose) over at least 20 minutes per dose, the second dose 12 hours after the first, and the third dose 24 hours after the second. If echocardiography revealed a patent ductus after these three doses, one to three additional doses were administered at 24 hours intervals guided by echocardiographic examinations after each additional dose. No additional pharmacological ductus treatment was administered. Surgical ligation was carried out if signs of a hemodynamically significant ductus arteriosus persisted after pharmacological treatment. Before discharge a clinical assessment of ductus arteriosus was made and additional echocardiographic examination performed if indicated.

### 2.3. Concomitant Treatment

According to the policy at the unit, infants received mechanical ventilatory support immediately after birth and early surfactant (Curosurf, Nycomed International, Zürich, Switzerland) administration (100 mg/kg) if signs of respiratory insufficiency were detected. Otherwise early nasal continuous positive airway pressure (CPAP) therapy was instituted. HFOV was used as rescue treatment.

Guideline fluid volumes were 90–100 mL/kg during the first day of life, adding 10 mL/kg/day each day during the first week for infants with a GA of 24 weeks or less, and 80–90 mL/kg, adding 10 mL/kg/day for infants with a GA of 25 to 27 weeks. Fluid intake was further individually adjusted, guided by weight loss, urinary output, and serum sodium concentration. Volume substitution and inotropic drugs were used restrictively and only when combinations of low mean arterial blood pressure and low micturition were detected. Bacterial cultures were taken and intravenous administration of antibiotics initiated if clinical suspicion of bacterial septicemia arose.

### 2.4. Perinatal Characteristics and Outcome at Discharge

Medical records were retrospectively assessed for information regarding GA, birth weight, gender, preeclampsia, antenatal steroids, Caesarean section, surfactant administration and Apgar-scores, and death. Nursing flow charts from the neonatal intensive care unit were assessed for details about PDA treatment, including information about ventilatory settings and fluid administration.

### 2.5. Echocardiography

Each infant's last echocardiographic examination before treatment start was reassessed for this study by a single cardiologist (A. Jonzon), who was blinded to treatment results. Maximal ductal flow velocity was assessed from the parasternal short axis view with pulsed and continuous Doppler directly in line with the ductal flow and ductal diameter was measured at the ductus narrowest inner dimension from the same position with and without color Doppler. Left atrium to aortic root ratio was measured in M-mode from the parasternal long axis.

The first follow-up echocardiography after treatment defined successful (Closed group) or failed (Persistent group) ductal closure. The PDA was considered closed if no ductal flow could be found with color Doppler.

### 2.6. Statistical Analysis

Data for each group is presented below as median values and range or number and percentage. Statistical analyses were conducted with SPSS Statistics 18 for Windows (SPSS, Inc., Chicago, Illinois, USA). The Mann-Whitney test was used to compare non-parametric continuous data and the Fisher's exact test was used to compare categorical data. All *P* values presented are two-tailed and a *P* < 0.05 was considered statistically significant. Multivariable logistic regression was performed to assess the individual influence of predictive factors on the proportion of ductal closure. Factors previously found to affect ductal closure during treatment (GA, preeclampsia, antenatal glucocorticoid administration, time of treatment start) were included in the analyses together with maximal ductal shunt velocity adjusted for the second power of ductal diameter and the time of echocardiography. The adjustment for second power of ductal diameter was made to assess whether flow velocity was independent of ductal diameter according to the Hagen-Poiseuille equation.

## 3. Results

Between January 2006 and December 2009, a total of 130 infants were born at 22–27 weeks of GA at Uppsala University Children's Hospital. Fifty-six infants received pharmacological treatment for patency of the ductus arteriosus and 6 infants received primary surgical treatment because of contraindications for pharmacological treatment before discharge from the same neonatal unit. In 18 infants the PDA closed spontaneously without treatment, 13 died and 37 did not receive pharmacological treatment before discharge because of either contraindications for treatment or a PDA not considered haemodynamically significant. Out of the 56 pharmacologically treated infants, 29 (52%) showed successful PDA closure (Closed group) and 27 (48%) failed to close (Persistent group).

### 3.1. Perinatal Characteristics

All observed perinatal characteristics were similar in the two groups with the exception of GA, which was higher in the Closed group (Table 1). No infant had been exposed to antenatal indomethacin. Median Apgar-scores at one, five, and ten minutes were 5, 7, and 9 in the Closed group and 5, 8, and 9 in the Persistent group (*P* = 0.980, 0.807, and 0.773, resp.).

### 3.2. Echocardiography

All infants had a predominately left to right ductal flow. Besides a higher maximal ductal flow velocity in the Closed group compared to the Persistent group, no other differences in echocardiographic parameters or ventilator characteristics were observed at the time of the last echocardiographic examination before treatment (Table 2).

### 3.3. Pharmacological PDA and Concomitant Treatment Characteristics

No major differences in treatment characteristics or fluid intake during treatment were observed between the two groups studied (Table 3). All studied infants treated for PDA had been given indomethacin except eight, who received treatment with ibuprofen alone (Pedea, Orphan Europe SARL, Paris La Défense, France, 5 mg/mL, first dose 10 mg/kg/dose and 5 mg/kg/dose 24 and 48 hours after first dose) and two infants who received both indomethacin and ibuprofen due to shortage of indomethacin during part of the studied period. Two infants in each group received only two doses of indomethacin (*P* = 1.000) because of contraindications for treatment. In three infants in the Persistent group, treatment had been initiated late after birth at 20, 24, and 40 days, respectively.

Multivariate logistical regression analysis for factors previously found to affect ductal closure during pharmacological PDA closure and maximal ductal shunt flow velocity (adjusted for squared ductal diameter and for time of echocardiography) still showed an association between higher maximal ductal flow velocity and ductal closure (Table 4).

### 3.4. Outcome at Discharge

Three infants in the Closed group had not undergone post-treatment echocardiography before discharge, but were considered clinically closed. They were discharged in good condition and have not been subjected to any further examination or treatment for ductus arteriosus since then. In the Persistent group, eleven infants (41%) were subjected to surgery after follow up echocardiography, one infant (4%) received a second course of ibuprofen at a regional hospital, three infants (11%) died with an open ductus, five infants (19%) had spontaneous closure of their PDA at the time of echocardiographic examination before discharge, and seven infants (26%) were discharged with an open ductus. Three infants (10%) in the Closed group had reopened PDAs since the first echocardiographic examination after treatment when repeatedly examined at 10, 23, and 28 days after treatment, respectively.

Three infants (10%) in the Closed group and 4 (15%) in the Persistent group died (*P* = 0.700) at a median of 38 (range 22–42) and 17 (range 6–166) days (*P* = 0.480), but no death was related to patency of the ductus arteriosus.

Using the Swedish Perinatal Quality Register (a national register for quality control of neonatal care) and nursing flow charts from the neonatal intensive care unit, information regarding use of HFOV was collected. Medical records were retrospectively assessed for information regarding diagnosis of culture-proven episodes of sepsis in connection to PDA treatment, bronchopulmonary dysplasia (BPD, defined as a need for supplemental oxygen at 36 weeks' GA), periventricular leukomalacia (PVL), IVH (including grade), retinopathy of prematurity (ROP), and NEC.

Eleven infants in the Closed group and 17 infants in the Persistent group were on ventilator treatment at the start of PDA treatment (*P* = 0.108). Infants in the Closed group received CPAP therapy for a median of 46 (range 3–95) days and were on ventilator treatment for a median of 8 (range 0–65 days) whereas infants in the Persistent group received CPAP therapy for a median of 42 (range 0–122) days and were on ventilator treatment for a median of 23 (range 0–100) days (*P* = 0.533 and 0.100, respectively). Four infants (14%) in the Closed group and seven infants (26%) in the Persistent group had undergone HFOV (*P* = 0.322), with a median of nine days (range 7–30) in the Closed group and four days (range 1–11) in the Persistent group (*P* = 0.037). None of the infants in the Closed group and two (7%) infants in the Persistent group had undergone HFOV before or during pharmacological treatment for PDA (*P* = 0.228). None of the infants in the Closed group and two (7%) infants in the Persistent group had undergone HFOV before or during pharmacological treatment for PDA (*P* = 0.228). One (3%) versus two (7%) infants had culture proven episodes of sepsis in connection to PDA treatment (*P* = 0.605).

Twenty-nine (100%) versus 27 (100%) infants were diagnosed with RDS (*P* = 1.000), 15 (52%) versus 16 (59%) with BPD (*P* = 0.602), 3 (10%) versus 4 (15%) with PVL (*P* = 0.700), 5 (17%) versus 7 (26%) with IVH (*P* = 0.523), 15 (52%) versus 15 (56%) with ROP (*P* = 0.795) and 1 (3%) versus 4 (15%) with NEC (*P* = 0.185) in the Closed and Persistent groups, respectively. One infant in each group had IVH grade III-IV (*P* = 1.000), and all others were grade I-II.

## 4. Discussion

Our study shows that higher gestational age and maximal shunt velocity is associated with successful pharmacological PDA treatment in infants born at 22–27 weeks GA. No other factor was found to differ between infants whose PDA closed and infants whose PDA did not close during treatment. In a multivariable logistic regression analysis, including the factors GA, preeclampsia, prenatal steroids, age at treatment start, and maximal ductal flow velocity adjusted for ductal diameter, only maximal ductal flow velocity was found to be independently associated with ductal closure. Furthermore, our study could not confirm any significant difference in outcome between infants whose PDA did or did not close during treatment.

Although the effect of ductal flow velocity on ductal closure has previously been noted in a more mature cohort of newborn infants, the mechanisms behind it are not extensively studied [14, 15, 20]. Assuming the ductus arteriosus to resemble a cylindrical pipe, the Hagen-Poiseuille equation states that the flow velocity in the ductus arteriosus is proportional to the pressure gradient between the systemic to pulmonary circulation and to the second power of the ductal diameter while it is inversely proportional to the blood viscosity and the ductal length [21]. In one previous study, the difference in maximal ductal flow velocity between infants whose ductus did or did not close has been suggested to be attributed to a difference in pulmonary arterial pressure [14]. In our study the influence of maximal ductal flow velocity was independent of the second power of the ductal diameter. Due to the retrospective design of our study we did not have the possibility to measure and adjust for blood viscosity or ductal length and we could only obtain data on the systolic blood pressure measured by arterial catheter at the time of echocardiography from 21 infants in the Closed group and 15 infants in the Persistent group. The uniformity in the measured systolic blood pressures between the two groups suggests however that a difference in the systemic to pulmonary circulation pressure gradient likely reflects a higher pulmonary arterial pressure in the Persistent group.

Pulmonary vascular resistance normally decreases rapidly with the start of ventilation and oxygenation after birth, reversing the fetal right to left flow through the ductus arteriosus and foramen ovale [22]. The normal physiological ductus arteriosus closure occurs in two stages, where the initial contraction of the vessel is a response to the decrease in pulmonary vascular resistance and pressure, an increase in arterial oxygen pressure, a decrease in circulating prostaglandin E2 (PGE2), and a decrease in PGE2-receptors in the ductal wall [19, 23–25]. Besides the vascular effect of a higher pulmonary pressure on the ductus arteriosus, the resulting lower blood flow and lower oxygen exposure of the ductus would therefore prevent an effective closing with pharmacological treatment. The previous finding of RDS as a risk factor for failure of pharmacological treatment of PDA underlines the close connection between ventilation, pulmonary circulation and the existence of a PDA [16, 18]. The lack of any major difference in ventilatory parameters and FiO_2_ between the Closed and Persistent groups in our study could indicate that ductal flow may be more sensitive to assess pulmonary vascular resistance at this early stage of life in extremely preterm infants.

Our study is limited by its retrospective design, the exclusion of a number of infants with PDA that were not treated before discharge, and the use of both indomethacin and ibuprofen during the study period. However, the two groups were well balanced and the study had the advantage of reflecting clinical treatment decisions based on strict guidelines.

## 5. Conclusion

In summary this study indicates that the maximal ductal flow velocity, independently of ductal diameter, is associated with successful treatment of PDA in extremely preterm infants. Pre-treatment echocardiographic maximal ductal flow velocity could possibly be used to assess the chances for treatment success in individual infants, but more information on the reliability of this parameter is needed.

## Figures and Tables

**Table 1:** Perinatal characteristics.

	Ductus closed (*n* = 29)	Ductus persistent (*n* = 27)	*P*
Gestational age, weeks (range)	25^+5^ (22^+2^–27^+4^)	24^+3^ (22^+3^–27^+4^)	**0.047**
Birth weight, grams (range)	718 (432–1217)	595 (440–1052)	0.363
Male gender (%)	20 (69)	14 (52)	0.274
Preeclampsia (%)	5 (17)	5 (19)	1.000
Antenatal steroids (%)	23 (79)	22 (81)	1.000
Cesarean section (%)	17 (59)	15 (56)	1.000
Surfactant (%)	28 (97)	26 (96)	1.000

**Table 2:** Characteristics at time of echocardiography.

	Ductus closed (*n* = 29)	Ductus persistent (*n* = 27)	*P*
Age at echocardiography, days (range)	2 (0–7)	2 (0–33)	0.079
Ventilator (%)	10 (34)	16 (59)	0.108
Ventilator MAP, cmH_2_O (range)	8 (6–14)	9 (7–12)	0.220
CPAP (%)	19 (66)	11 (41)	0.108
CPAP pressure, cmH_2_O (range)	5 (4–7)	5 (3–7)	0.618
Fraction of inspired oxygen, % (range)	25 (21–52)	27 (21–42)	0.848
Systolic blood pressure^1^, mmHg (range)	47 (37–62)	47 (35–84)	0.987
Ductal diameter, mm (range)	1.7 (0.9–3.0)	1.8 (1.0–3.0)	0.399
Maximal ductal flow velocity, m/s (range)	1.6 (0.5–2.7)	1.1 (0.7–2.9)	**0.023**
LA/Ao (range)	1.5 (1.2–2.8)	1.7 (1.1–3.5)	0.198

^1^21 versus 15 infants had an arterial catheter which enabled blood pressure measurements.

**Table 3:** Treatment characteristics.

	Ductus closed (*n* = 29)	Ductus persistent (*n* = 27)	*P*
Age at treatment start, days (range)	3 (1–8)	3 (1–40)	0.117
Indomethacin (%)	26 (90)	22 (82)	0.462
Ibuprofen^1^ (%)	4 (14)	6 (22)	0.497
Change in weight, % (range)	1 (−10–9)	4 (−12–12)	0.090
Fluid intake, mL/kg/day (range)	134 (98–168)	139 (111–203)	0.354
Part IV, % (range)	59 (0–85)	43 (0–89)	0.468
Urine output, mL/kg/h (range)	2.1 (0.5–4.2)	2.2 (0.4–4.9)	0.594

^1^1 versus 1 infants received treatment with both indomethacin and ibuprofen during the same course.

**Table 4:** Multivariable analysis for ductal closure.

	OR (95% CI)	*P*
Gestational age^1^	1.45 (0.93–2.25)	0.103
Preeclampsia	0.78 (0.11–5.60)	0.807
Antenatal steroids	0.83 (0.17–4.04)	0.817
Age at treatment start^2^, days	0.82 (0.60–1.12)	0.213
Maximal ductal flow velocity^3^, m/s	3.04 (1.01–9.22)	**0.049**

^1^OR for every 1 week increase.

^2^OR for every 1 day increase.

^3^OR for every 1 m/s increase, adjusted for age at echocardiography and squared ductal diameter.
